# Supplementary material for: Prevalence of Respiratory Infections during the 2018–2020 Period in the Paediatric Population of Primary Care Centres in Central Catalonia
Source: Healthcare (Basel). 2023 Apr 27;11(9):1252. doi: 10.3390/healthcare11091252 (PMC10178082; doi:10.3390/healthcare11091252)
Supplement: Supplementary file 1 [file healthcare-11-01252-s001.zip › healthcare-2296683-supplementary.pdf]

healthcare

Table S1. Prevalence of respiratory infections by sex

	Male						Female					
	2018		2019		2020		2018		2019		2020	
	n (%)	CI 95% (%)	n (%)	CI 95% (%) / P-value	n (%)	CI 95% (%) / P-value	n (%)	CI 95% (%)	n (%)	CI 95% (%) / P-value	n (%)	CI 95% (%) / P-value
**Acute tonsillitis**	2454 (7,49)	(7.21;7.78)	2083 (6,35)	(6.09;6.62)	812 (2,53)	(2.36;2.7)	2240 (7,29)	(7.01;7.59)	1889 (6,11)	(5.85;6.39)	730 (2,42)	(2.25;2.6)
Variation 2019 vs 2018			-371 (-15,12)	<0,001					-351 (-15,67)	<0,001		
Variation 2020 vs 2019					-1271 (-61,02)	<0,001					-1159 (-61,36)	<0,001
**Streptococcal pharyngitis**	943 (2,88)	(2.7;3.07)	1448 (4,42)	(4.2;4.65)	395 (1,23)	(1.11;1.36)	952 (3,1)	(2.91;3.3)	1370 (4,43)	(4.21;4.67)	436 (1,44)	(1.31;1.59)
Variation 2019 vs 2018			505 (53,55)	<0,001					418 (43,91)	<0,001		
Variation 2020 vs 2019					-1053 (-72,72)	<0,001					-934 (-68,18)	<0,001
**Bronchiolitis**	558 (1,7)	(1.57;1.85)	596 (1,82)	(1.68;1.97)	161 (0,5)	(0.43;0.59)	377 (1,23)	(1.11;1.36)	445 (1,44)	(1.31;1.58)	135 (0,45)	(0.38;0.53)
Variation 2019 vs 2018			38 (6,81)	0,278					68 (18,04)	0,023		
Variation 2020 vs 2019					-435 (-72,99)	<0,001					-310 (-69,66)	<0,001
**Bronchitis**	2262 (6,9)	(6.63;7.19)	2058 (6,28)	(6.02;6.55)	887 (2,76)	(2.58;2.94)	1775 (5,78)	(5.52;6.05)	1589 (5,14)	(4.9;5.4)	630 (2,08)	(1.93;2.25)
Variation 2019 vs 2018			-204 (-9,02)	0,001					-186 (-10,48)	0,001		
Variation 2020 vs 2019					-1171 (-56,9)	<0,001					-959 (-60,35)	<0,001
**COVID**	-	-	-	-	1144 (3,56)	(3.36;3.77)	-	-	-	-	1092 (3,61)	(3.41;3.83)
**Stomatitis**	416 (1,27)	(1.15;1.4)	309 (0,94)	(0.84;1.05)	59 (0,18)	(0.14;0.24)	367 (1,2)	(1.08;1.32)	253 (0,82)	(0.72;0.93)	44 (0,15)	(0.11;0.2)
Variation 2019 vs 2018			-107 (-25,72)	<0,001					-114 (-31,06)	<0,001		
Variation 2020 vs 2019					-250 (-80,91)	<0,001					-209 (-82,61)	<0,001
**Pharyngitis**	2262 (6,9)	(6.63;7.19)	2265 (6,91)	(6.64;7.19)	995 (3,09)	(2.91;3.29)	2254 (7,34)	(7.05;7.64)	2180 (7,06)	(6.77;7.35)	943 (3,12)	(2.93;3.32)
Variation 2019 vs 2018			3 (0,13)	0,999					-74 (-3,28)	0,180		
Variation 2020 vs 2019					-1270 (-56,07)	<0,001					-1237 (-56,74)	<0,001
**Flu- like syndrome**	933 (2,85)	(2.67;3.04)	934 (2,85)	(2.67;3.04)	1268 (3,94)	(3.73;4.16)	859 (2,8)	(2.62;2.99)	803 (2,6)	(2.43;2.78)	1149 (3,8)	(3.59;4.03)
Variation 2019 vs 2018			1 (0,11)	1,000					-56 (-6,52)	0,136		
Variation 2020 vs 2019					334 (35,76)	<0,001					346 (43,09)	<0,001
**Laryngotracheitis**	1291 (3,94)	(3.73;4.16)	1600 (4,88)	(4.65;5.12)	652 (2,03)	(1.88;2.19)	942 (3,07)	(2.88;3.27)	1182 (3,83)	(3.62;4.05)	469 (1,55)	(1.42;1.7)
Variation 2019 vs 2018			309 (23,93)	<0,001					240 (25,48)	<0,001		
Variation 2020 vs 2019					-948 (-59,25)	<0,001					-713 (-60,32)	<0,001
**Otitis media**	3449 (10,53)	(10.2;10.87)	3369 (10,27)	(9.95;10.61)	1482 (4,61)	(4.38;4.84)	3255 (10,6)	(10.26;10.95)	3072 (9,94)	(9.61;10.28)	1418 (4,69)	(4.46;4.94)
Variation 2019 vs 2018			-80 (-2,32)	0,295					-183 (-5,62)	0,008		
Variation 2020 vs 2019					-1887 (-56,01)	<0,001					-1654 (-53,84)	<0,001
**Pneumonia**	363 (1,11)	(1;1.23)	351 (1,07)	(0.96;1.19)	147 (0,46)	(0.39;0.54)	326 (1,06)	(0.95;1.18)	353 (1,14)	(1.03;1.27)	148 (0,49)	(0.42;0.58)
Variation 2019 vs 2018			-12 (-3,31)	0,671					27 (8,28)	0,345		
Variation 2020 vs 2019					-204 (-58,12)	<0,001					-205 (-58,07)	<0,001
**Common cold**	8113 (24,76)	(24.3;25.24)	7341 (22,39)	(21.94;22.84)	4423 (13,75)	(13.38;14.14)	7681 (25,01)	(24.53;25.5)	6949 (22,49)	(22.03;22.97)	4002 (13,24)	(12.86;13.63)
Variation 2019 vs 2018			-772 (-9,52)	<0,001					-732 (-9,53)	<0,001		
Variation 2020 vs 2019					-2918 (-39,75)	<0,001					-2947 (-42,41)	<0,001

**Actue sinusitis**	181 (0,55)	(0.48;0.64)	182 (0,56)	(0.48;0.64)	122 (0,38)	(0.32;0.45)	213 (0,69)	(0.61;0.79)	183 (0,59)	(0.51;0.69)	106 (0,35)	(0.29;0.43)
Variation 2019 vs 2018			1 (0,55)	1,000					-30 (-14,08)	0,128		
Variation 2020 vs 2019					-60 (-32,97)	<0,001					-77 (-42,08)	<0,001
